# Determining clinical course of diffuse large B-cell lymphoma using targeted transcriptome and machine learning algorithms

**DOI:** 10.1038/s41408-022-00617-5

**Published:** 2022-02-01

**Authors:** Maher Albitar, Hong Zhang, Andre Goy, Zijun Y. Xu-Monette, Govind Bhagat, Carlo Visco, Alexandar Tzankov, Xiaosheng Fang, Feng Zhu, Karen Dybkaer, April Chiu, Wayne Tam, Youli Zu, Eric D. Hsi, Fredrick B. Hagemeister, Jooryung Huh, Maurilio Ponzoni, Andrés J. M. Ferreri, Michael B. Møller, Benjamin M. Parsons, J. Han van Krieken, Miguel A. Piris, Jane N. Winter, Yong Li, Bing Xu, Ken H. Young

**Affiliations:** 1 Genomic Testing Cooperative, LCA, Irvine, CA 92618 USA; 2 John Theurer Cancer Center at Hackensack University Medical Center, Hackensack, NJ 07601 USA; 3 Duke University Medical Center, Durham, NC 27710 USA; 4 Columbia University Medical Center, New York, NY 10027 USA; 5 University of Verona, 37129 Verona, Italy; 6 Institute of Pathology, University Hospital Basel, 4054 Basel, Switzerland; 7 Aalborg University Hospital, Aalborg, 5000–5270 Denmark; 8 Mayo Clinic, Rochester, MN 55905 USA; 9 Weill Medical College of Cornell University, New York, NY 10065 USA; 10 The Methodist Hospital, Houston, TX 77030 USA; 11 Wake Forest University Medical Center, Winston-Salem, NC 77055 USA; 12 The University of Texas MD Anderson Cancer Center, Houston, TX 22030 USA; 13 Asan Medical Center, Ulsan University College of Medicine, Seoul, 05505 Korea; 14 San Raffaele H. Scientific Institute, 20132 Milan, Italy; 15 Odense University Hospital, Odense, 5000–5270 Denmark; 16 Gundersen Lutheran Health System, La Crosse, WI 54601 USA; 17 Radboud University Nijmegen Medical Centre, 6500 Nijmegen, Netherlands; 18 Hospital Universitario Marqués de Valdecilla, 39008 Santander, Spain; 19 Feinberg School of Medicine, Northwestern University, Chicago, IL 60611 USA; 20 Baylor College of Medicine, Houston, TX 77030 USA; 21 The First Affiliated Hospital of Xiamen University, 361004 Xiamen, Fujian China; 22 Duke Cancer Institute, Durham, NC 27710 USA

**Keywords:** Cancer, Translational research

## Abstract

Multiple studies have demonstrated that diffuse large B-cell lymphoma (DLBCL) can be divided into subgroups based on their biology; however, these biological subgroups overlap clinically. Using machine learning, we developed an approach to stratify patients with DLBCL into four subgroups based on survival characteristics. This approach uses data from the targeted transcriptome to predict these survival subgroups. Using the expression levels of 180 genes, our model reliably predicted the four survival subgroups and was validated using independent groups of patients. Multivariate analysis showed that this patient stratification strategy encompasses various biological characteristics of DLBCL, and only TP53 mutations remained an independent prognostic biomarker. This novel approach for stratifying patients with DLBCL, based on the clinical outcome of rituximab, cyclophosphamide, doxorubicin, vincristine, and prednisone therapy, can be used to identify patients who may not respond well to these types of therapy, but would otherwise benefit from alternative therapy and clinical trials.

## Introduction

Diffuse large B-cell lymphoma (DLBCL) is the most common subtype of lymphoma. However, this disease is heterogeneous [1–4], i.e., its outcome and course may vary significantly between patients [1]. More than 60% of patients with DLBCL can be cured with rituximab, cyclophosphamide, doxorubicin, vincristine, and prednisone (R-CHOP) treatment [1]. Multiple new combinations of therapeutic strategies, including cell therapy, are being tested to improve survival, especially in patients who may not respond to the standard cyclophosphamide, doxorubicin, vincristine, and prednisone therapy [5]. Considering the known heterogeneity of DLBCL, a single therapeutic approach is unlikely to work with all patients with DLBCL [1]. Therefore, multiple approaches have been used to subclassify DLBCL into various subgroups based on biological characteristics. The earliest subclassification was based on expression profiling using microarrays [6–9]. This classification divides DLBCL into two major groups, namely germinal center B-cell-like (GCB) and activated B-cell-like (ABC) DLBCL, based on the cell of origin (COO). In this classification, 15% of DLBCL cases were classified into the other group. Based on subsequent refining of this classification, the GenClass algorithm was developed. In this algorithm, genetic abnormalities are divided into four groups: *MYD88* and *CD79B* mutations (MCD), *BCL6* fusions and *NOTCH2* mutations (BN2), *NOTCH1* mutations (N1), and *EZH2* mutations and *BCL2* translocations (EZB); nevertheless, this algorithm can classify only 54% of DLBCL cases. To cover more cases, this algorithm was later extended as the LymphGen algorithm which divides genetic abnormalities into seven groups: MCD, N1, and BN2, as in the GenClass algorithm; MYC-negative and MYC-positive EZB; TP53 abnormality (A53) and mutations in TET2, P2RY8, or GSK1 (ST2) [6].

Using mutation profiling and chromosomal structural abnormalities (chromosomal gains and losses), Chapuy et al. classified DLBCL into five subgroups [9]. Recent FISH tests (double or triple hit) demonstrated that the rearrangement of MYC (Avian Myelocytomatosis Viral Oncogene Homolog) when co-present with BCL2, BCL6, or both leads to a significantly more aggressive DLBCL, making R-CHOP ineffective [10, 11].

While existing strategies for the subclassification of DLBCLs can distinguish biologically distinct subgroups of DLBCLs, they cannot effectively predict the overall survival or progression-free survival and their distinction performance is not satisfactory [1]. Furthermore, the clinical implementation of these classifications in routine laboratory testing is complicated by the need for performing whole-exome sequencing.

We rationalized that chromosomal structural analysis and mutation profiling eventually lead to changes in RNA profiling and activation or suppression of various pathways through relative RNA changes; thus, the RNA-based classification of DLBCL might be more practical. RNA quantification by next-generation sequencing (NGS) has numerous advantages over quantification methods based on microarrays and hybridization. RNA quantification by NGS is more specific and reproducible and can be performed reliably on formalin-fixed paraffin-embedded (FFPE) tissue. Furthermore, targeted RNA sequencing has the potential to be used in clinical testing because it is easier to manage and more cost-effective as a routine clinical test than traditional methods.

In this study, we developed a DLBCL classification strategy for predicting clinical outcomes using targeted RNA sequencing combined with machine learning algorithms. The developed strategy classifies patients with DLBCL into subgroups based on the clinical course of their disease. To focus on survival, we first used machine learning and divided the patients into subgroups based on their overall survival. We used modified Bayesian statistics to select genes that can predict various survival groups, and then validated these biomarkers using an independent set of cases.

## Results

### Naïve model for the survival of patients with DLBCL

**Fig. 1:**
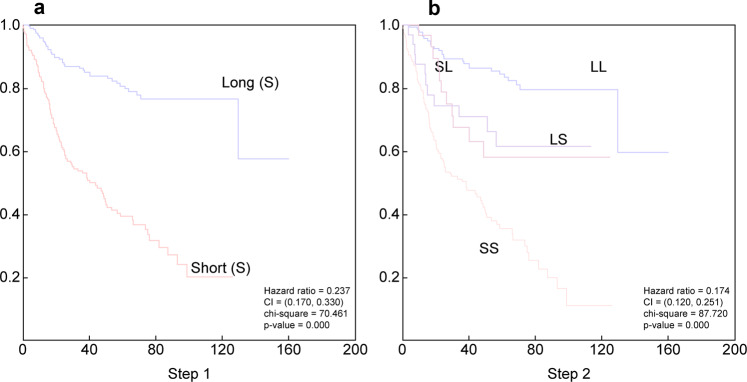
Prediction of patient survival using supervised machine learning without biomarkers (379 cases). **a** Survival when divided into two group. **b** Survival when each of the previous group is further divided into two groups. CI confidence interval.

Instead of defining biomarkers and then evaluating clinical behavior based on specific markers, we first grouped patients based on their survival, and then used biomarkers to predict these groups. We used a machine learning method to analyze the survival data. For a case that is not censored, the survival time is known. However, for a censored case, we do not know the exact survival time. Therefore, the censored data cannot be used as training data for supervised learning machine algorithms because they do not have a target value. However, omitting the censored data would reduce the sample size. Therefore, we used a machine learning approach to predict the survival of censored patients. First, we divided the patients into two groups: short survival (S) and long survival (L) (Fig. 1a). The hazard ratio was 0.237 (confidence interval: 0.170–0.330), and *P*-value < 0.00001. The survival of the patients in each group was not homogeneous. To refine this model, we used the same approach and divided the patients in each group into two subgroups, generating four groups: long survival in the long group (LL), short survival in the long group (LS), long survival in the short group (SL), and short survival in the short group (SS) (Fig. 1b). The hazard ratio for this model was 0.174 (confidence interval: 0.120–0.251), and *P*-value < 0.0001.

### Selecting biomarkers for predicting survival groups using machine learning

After defining the survival groups, a machine learning algorithm was developed to predict the survival time using the expression data of 1408 genes from the NGS data. We developed a generalized naïve Bayesian classifier by applying a geometric mean to the likelihood product to eliminate underflow. Through this approach, we ranked the 1408 biomarkers for predicting each survival group. However, the use of many biomarkers leads to overfitting. To reduce the effects of noise and avoid overfitting, we employed 12-step cross-validation to obtain a robust measure. For an individual gene, a generalized naïve Bayesian classifier was constructed on the training of one of the 12 subsets and tested on the other 11 testing subsets. This allowed us to limit the prediction process to 60 genes for each separation step. Sixty genes were used to predict S and L; the second set of 60 genes was used to predict LL and LS, and the third set of 60 genes was used to predict SL and SS. Table S1 lists the selected genes in each step. There was very little overlap among the three groups of biomarkers. As shown in Fig. 1b, the overall survival rates of LS and SL were similar. However, completely different sets of genes were used for selecting each group. This indicates that even though these two groups have similar clinical courses, they are completely biologically different. This reflects the significant heterogeneity of DLBCL.

### Validation of the survival model and selected biomarkers

**Fig. 2:**
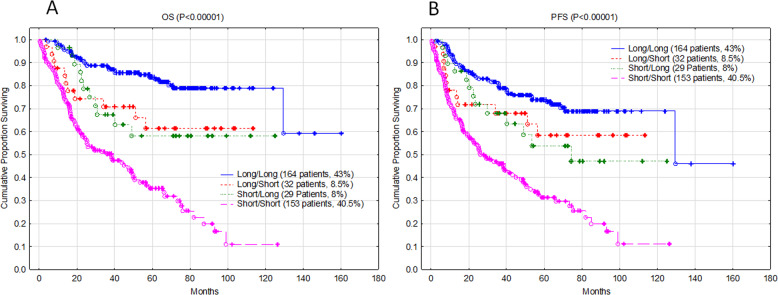
Validation of the machine learning models used for survival grouping and selection of biomarkers. **a** Actual overall survival (OS) and **b** progression-free survival (PFS) of the four groups as predicted by the selected biomarkers.

After building a survival model solely based on the survival data, then selecting biomarkers that can specifically correlate with these survival groups, we tested if these biomarkers could stratify patients accordingly. Using the selected biomarkers, we first classified the patients in the original set (379 patients) into LL, LS, SL, and SS groups and then evaluated the survival pattern of these groups. The characteristics of these patients are listed in Table S2. This group of patients included 239 (63%) diagnosed with a nodal disease and 140 (37%) with extranodal disease. The international prognostic index (IPI) was >2 in 141 (37%) patients and the rest of the patients (63%) had IPI ≤ 2. The Eastern Cooperative Oncology Group performance status (ECOG) score was >1 in 60 (16%) patients and <2 in 319 (84%) of patients. Of these patients, 210 (55%) were males. As shown in Fig. 2A, the selected biomarkers predicted survival as expected in the overall survival groups prior to biomarker selection. The same was true for the predicted progression-free survival (Fig. 2B).

**Fig. 3:**
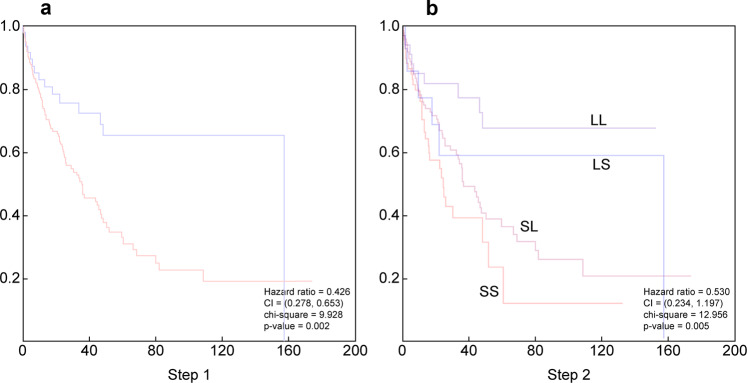
Validation of the machine learning models using independent set of 247 extranodal DLBCL samples. **a** Overallsurvival using two groups model and **b** overall survival using four groups model.

For additional validation of the system, we used the selected biomarkers to classify a completely new set of 247 samples of patients presented with extranodal DLBCL. As shown in Fig. 3, these selected biomarkers successfully predicted the overall survival in this group of patients when they were divided into two groups using the first set of biomarkers (Table S1) with an HR of 0.26 (confidence interval: 0.278–0.653, *P*-value = 0.002), as well as when they were divided into four groups using the three sets of biomarkers with an HR of 0.530 (confidence interval: 0.234–1.197, *P* = 0.005) (Fig. 3). As expected, extranodal DLBCL leads to overall shorter survival and more aggressive disease. To further test the reliability of this modeling system, we combined the two groups of patients (626 patients) and used two-thirds for building the model and one-third for testing. The overall model remained substantially the same, especially in the testing group. The testing group clearly shows two groups of patients with intermediate survival, but significantly different biological backgrounds (Fig. S1).

### Correlation with cell of origin (COO) classification and other clinical prognostic markers

**Fig. 4:**
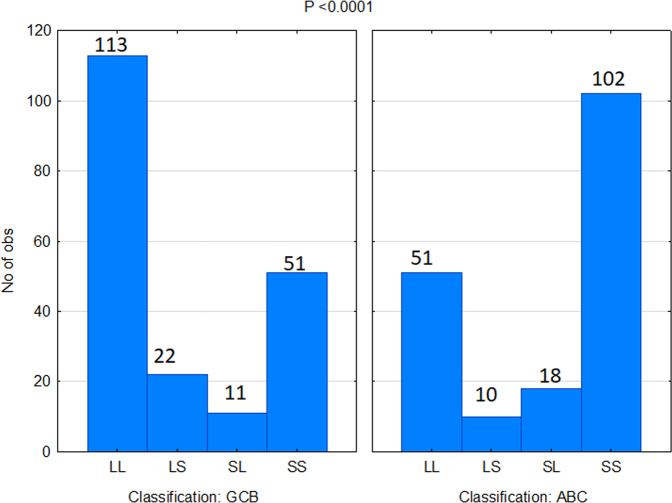
Correlation between survival groups and cell of origin classification. The left panel shows that majority of patients classified as germinal center B-cell-like (GCB) are classified as having long survival (LL) on the survival model. The right panel shows that majority of patients classified as activated B-cell-like (ABC) are classified as having short survival (SS).

As previously mentioned, all 379 patients were classified as cells of origin. We evaluated the prevalence of ABC and GCB groups in our survival groups. The majority of GCB cases had a good prognosis (LL and LS; *P* < 0.0001) (Fig. 4). Furthermore, although the LS and SL groups showed similar overall survival, there were significantly more GCB cases in the LS group than in the SL group (*P* = 0.016). This also confirms that, despite having similar outcomes, the LS and SL groups are biologically different.

In Cox proportional hazard regression multivariate model incorporating the survival classification with COO and the IPI (IPI ≤ 2 vs IPI > 2), survival classification and IPI were the only independent predictors of survival. In this model, COO was no longer a predictor of survival (Table 1). In a multivariate model incorporating age without IPI, age was a significant independent predictor of survival (*P* = 0.01). Poor survival subgroup (SS) had a significantly (*P* = 0.01) higher percentage of patients at age above 60 (Fig. S2). This raises the possibility that age and possible death from causes other than lymphoma—and not only biology-contribute to the poor survival in the SS subgroup.

**Table 1:** Multivariate survival analysis.

*N* = 379	Beta	Standard error	Beta/coefficient		*p*	Hazard ratio	Hazard ratio	
			95% lower	95% upper			95% lower	95% upper
Covariates: survival groups, cell of origin, and IPI (>2)								
Survival classification	0.55	0.07	0.40	0.69	0.000000	1.73	1.49	2.00
GCB vs ABC	−0.03	0.18	−0.37	0.32	0.869145	0.97	0.69	1.37
IPI	0.88	0.17	0.54	1.21	0.000000	2.41	1.72	3.36
Covariates: survival groups, IPI (>2), cell of origin, and TP53 mutation								
Survival classification	0.53	0.08	0.39	0.68	0.000000	1.71	1.47	1.98
IPI	0.84	0.17	0.50	1.18	0.000001	2.32	1.66	3.24
COO classification	0.04	0.18	−0.31	0.40	0.816543	1.04	0.73	1.49
Mute.TP53	0.37	0.19	0.00	0.73	0.048156	1.44	1.00	2.08
Covariates: survival groups, IPI (>2), cell of origin, mutations in MYD88, CD79B, and TP53 mutation								
Survival classification	0.54	0.08	0.40	0.69	0.000000	1.72	1.49	2.00
IPI	0.87	0.17	0.53	1.21	0.000000	2.39	1.71	3.36
COO classification	0.13	0.19	−0.24	0.50	0.491261	1.14	0.79	1.64
Mute.MYD88	−0.45	0.22	−0.88	−0.02	0.041843	0.64	0.41	0.98
Mute.CD79B	0.06	0.32	−0.55	0.68	0.841714	1.06	0.57	1.98
Mute. TP53	0.38	0.19	0.01	0.74	0.044687	1.46	1.01	2.10
Covariates: survival groups, IPI (>2), cell of origin, TP53 mutation, and MYC expression (above upper 25 percentile)								
Survival classification	0.54	0.08	0.39	0.69	0.000000	1.71	1.47	1.99
IPI	0.84	0.17	0.51	1.18	0.000001	2.32	1.66	3.24
Classification	0.04	0.18	−0.31	0.40	0.816151	1.04	0.73	1.49
Mute.TP53	0.37	0.19	0.00	0.74	0.048720	1.45	1.00	2.11
MYC U25%	−0.03	0.18	−0.39	0.33	0.878706	0.97	0.68	1.39
Covariates: survival groups, IPI (>2), cell of origin, TP53 mutation, and MYC expresion (continuous variable)								
Survival classification	0.55	0.08	0.41	0.70	0.000000	1.74	1.50	2.02
IPI	0.85	0.17	0.52	1.19	0.000001	2.35	1.68	3.28
Classification	0.03	0.18	−0.33	0.38	0.886603	1.03	0.72	1.46
Mute.TP53	0.41	0.19	0.04	0.78	0.028204	1.51	1.04	2.18
MYC	0.00	0.00	0.00	0.00	0.150307	1.00	1.00	1.00
Covariates: survival groups, IPI (>2), cell of origin, TP53 mutation, and expression of MYC and IRF4 (continuous)								
Survival classification	0.59	0.08	0.43	0.74	0.000000	1.80	1.54	2.09
IPI	0.85	0.17	0.51	1.18	0.000001	2.33	1.67	3.26
COO classification	0.21	0.21	−0.19	0.61	0.308746	1.23	0.82	1.84
Mute.TP53	0.43	0.19	0.06	0.80	0.022837	1.54	1.06	2.22
MYC mRNA	0.00	0.00	0.00	0.00	0.124518	1.00	1.00	1.00
IRF4 mRNA	0.00	0.00	0.00	0.00	0.066811	1.00	1.00	1.00

*GCB* germinal center B-cell-like, *ABC* activated B-cell-like, *COO* cell of origin, *MYC* Avian Myelocytomatosis Viral Oncogene Homolog, *IPI* Iinternational Prognostic Index, *ECOG*, Eastern Cooperative Oncology Group performance status.

### Correlation with TP53 mutation

Of the 379 DLBCL patients, 82 (22%) had TP53 mutations. As expected, patients with TP53 had significantly shorter survival rates (*p* = 0.0019). There were relatively more TP53 mutations in the short survival groups (*P* = 0.009) (Fig. S3). More importantly, in a multivariate model incorporating TP53 mutation with survival classification, IPI, and COO, TP53 mutations remained strong independent predictors of survival (Table 1).

### Correlation with MYD88 and CD79B mutations

Patients with MYD88 mutations were more common in the S group (*P* = 0.001) with aggressive DLBCL. However, there was no significant difference in the distribution of patients with CD79B mutations among the various survival groups (*P* = 0.49). In a multivariate model incorporating mutations in TP53, CD79B, and MYD88 along with COO, IPI, and survival classification, the mutation in CD79B was not a predictor of survival, but MYD88 was an independent predictor of better survival (*P* = 0.042), and TP53 mutation remained a predictor of worse survival (*P* = 0.045) (Table 1).

### Correlation with MYC overexpression

**Fig. 5:**
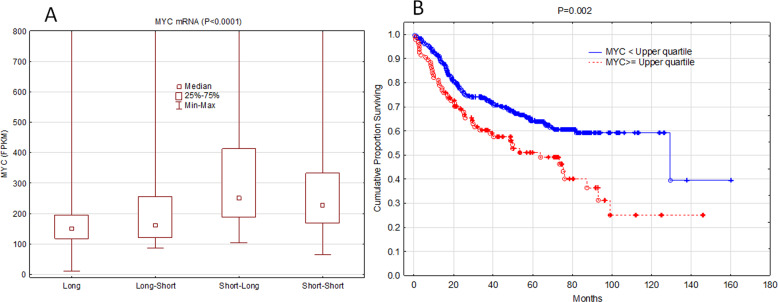
MYC overexpression as predictor of survival. **a** The levels of MYC mRNA in various survival groups. **b** Kaplan–Meier survival curves of patients based on MYC expression.

MYC expression was significantly higher in the S groups (*P* < 0.0001). Higher levels of MYC mRNA were detected in the SL group than in the LS group (*P* < 0.0001), although the two groups showed similar survival (Fig. 5A). Short survival was associated with high MYC expression when used as a continuous variable (*P* = 0.0019) or when patients were grouped as low vs. high based on the upper quartile (*P* = 0.0021) (Fig. 5B). However, in the multivariate model, MYC expression was not an independent predictor of survival, irrespective of whether it was used as a continuous and categorical (low vs. high) variable (Table 1).

### Correlation with IRF4 overexpression

IRF4 gene translocation is typically associated with overexpression [12–14]. Recent studies have shown that DLBCL with IRF4 translocation is less damaging. We investigated IRF4 RNA overexpression and correlated it with the survival groups, as predicted in our model. Significant overexpression of IRF4 mRNA was observed in the S group of patients (Fig. S4). As well as lower levels of MYC, the LS group had significantly lower levels of IRF4 mRNA than the SL group (*P* = 0.02), although there was no difference in survival between these two groups. In a multivariate model incorporating the survival groups, among COO, IPI, MYC, TP53, and IRF4 mRNA as continuous variables, IRF4 mRNA level was a borderline (*P* = 0.067) negative predictor of survival in this model (Table 1).

## Discussion

DLBCL is a heterogeneous disease with complex biological variations in the form of gene mutations, chromosomal structural abnormalities, chromosomal translocations, and microenvironment changes. Subclassification of DLBCL must account for changes in all these driving biological determinants. In principle, all these biological determinants lead to changes in the RNA levels of various genes in the tumor and microenvironment. Existing methods for the evaluation of the RNA expression and measurements of the RNA levels are highly reliable. In particular, NGS counts the number of RNA molecules without significant influence of hybridization or amplification artifacts [15]. Furthermore, targeted RNA sequencing and targeted transcriptome have a high dynamic range and can determine the biologically relevant genes and reduce the bias in the sequencing of the highly expressed genes effectively. Therefore, targeted RNA expression profiling by NGS can effectively subclassify DLBCLs by encompassing all biological determinants of clinical behavior and outcome.

However, the subclassification of disease must reflect its clinical behavior. This is complicated by the fact that clinical behavior may be influenced by the therapy selected. The current standard therapy for DLBCL is R-CHOP. To improve this therapy, patients should be classified based on the type of response or lack thereof to this standard therapy. This may allow us to predict the biomarkers that determine the type of response and target the biological pathways driving these biomarkers. This approach might reduce overfitting in the process of selecting biomarkers that predict various types of responses. In other words, instead of biomarkers predicting survival, it might be more relevant clinically to let survival predict biomarkers.

We followed this strategy to classify DLBCL. First, we developed an approach to predict the survival groups. We predicted the survival of censored patients using machine learning. Based on this, we divided the entire patient population into two L and S groups. In a tree model, we also divided the L group into LL and LS, and the S group into SL and SS groups; the HR in these groups was 0.174 (Fig. 1B). Then, we explored the ability of targeted RNA expression data generated from sequencing 1408 genes in predicting these survival groups using naïve Bayesian statistics. However, prediction using naïve Bayesian typically shows steep prediction distributions, making it difficult to compare values. Thus, we smoothed these distributions to facilitate a comparison between each biomarker, as described in the methods (Fig. S5). To avoid overfitting, we randomly divided the 378 patients into 12 different groups. We cross-validated the selected biomarkers among the 12 subgroups. This approach allowed us to select 60 biomarkers for the first set of survival subgroups (Fig. 1A) and 60 for each of the subsequent survival subgroups (Fig. 1B). Using these biomarkers, we classified the 378 patients accurately, as predicted by the machine learning algorithm (Fig. 2). To further validate these biomarkers, we used an independent group of 247 patients with extranodal DLBCL. As shown in Fig. 3A and B, these biomarkers efficiently predicted survival in the extranodal patients despite the shorter overall survival, as expected in this group of patients.

The classification based on survival correlated with COO classification, TP53 mutation status, MYC expression, and IRF4 expression. In the multivariate analysis using the survival 4 group model, only IPI and TP53 mutations were independent in predicting the prognosis (Table 1). MYD88 mutation was an independent predictor of good prognosis in multivariate analysis. This data shows that our genomic survival model provides important information on the clinical behavior of DLBCL that is independent of IPI and other prognostic indicators. Furthermore, this genomic classification defines specific genes (see Table S1) that are driving each of the survival groups’ defined models. Potentially this list of genes may provide useful clues for targeted therapy that can be tailored to each of the survival groups defined in this model.

These findings suggest that the subclassification of patients using survival is a reliable approach to define biologically different patients with DLBCL. In fact, although the LS and SL groups had similar survival, they had significantly different MYC and IRF4 levels. This supports our assumption that it is unrealistic to assume that one biomarker can define specific clinical behavior and that significant overlap between biomarkers exists in driving the biology of DLBCL.

The objective of this classification is to predict DLBCL patients who will not respond to R-CHOP so that they can be treated differently, or they can be entered into clinical trials. It may be easier to find a new successful therapeutic approach when patients with similar biology and clinical courses are treated in clinical trials with new therapeutic regimens. This subclassification of DLBCL can be automated through simple software that we developed and can predict the survival subgroup when fed with RNA sequencing data.

## Methods

### Patients

RNA sequencing using a targeted panel was performed on samples from 379 patients with de novo DLBCL and 247 patients with extranodal DLBCL. The samples from the 379 patients were used to establish the prognostic model, and those from the 247 patients were used for validation. All patients were treated with R-CHOP at 22 medical centers. The cases were organized and collected using the DLBCL Consortium Program, which was approved by the institutional review board of each participating medical center and conducted in accordance with the Declaration of Helsinki. The ethics committee waived the requirement for informed consent owing to the retrospective study design. Patients with transformed DLBCL, primary mediastinal large B-cell lymphoma, or primary cutaneous DLBCL were excluded.

### RNA library construction and sequencing

The Agencourt FormaPure Total 96-Prep Kit was used to extract DNA and RNA from the same FFPE tissue lysates using an automated KingFisher Flex following the protocols recommended by the manufacturers. Samples were selectively enriched for 1408 cancer-associated genes using reagents provided in the Illumina® TruSight® RNA Pan-Cancer Panel. cDNA was generated from the cleaved RNA fragments using random primers during the first and second-strand synthesis. Sequencing adapters were ligated to the resulting double-stranded cDNA fragments. The coding regions of the expressed genes were captured from this library using sequence-specific probes to create the final library. Sequencing was performed using an Illumina NextSeq 550 system platform. Ten million reads per sample in a single run were required, and the read length was 2 × 150 bp. The sequencing depth was 10×–1739× with a median of 41×. An expression profile was generated from the sequencing coverage profile of each individual sample using Cufflinks. Expression levels were measured as fragments per kilobase of transcript per million.

### Machine learning methods for survival analysis

We used a machine learning method to estimate the survival time of a censored patient, for which we did not know the survival time, using the Kaplan–Meier curve.

#### Theorem

Let $S(t)$ be the survival function and $f(t)$ be the probability density function of survival. For a censored case at time ${t}_{0}$, the conditionally expected survival time is

$${t}_{0}+\frac{1}{S({t}_{0})}{\int_{{t}_{0}}^{{{\infty }}}}S\left(t\right){dt}.$$

#### Proof

Given the censored time ${t}_{0}$, the conditional density function is

$$\frac{f(t)}{S({t}_{0})},\,t\ge {t}_{0},$$

and the expectation is

$$\begin{array}{ll}{\int_{{t}_{0}}^{{{\infty }}}}t\frac{f\left(t\right)}{S\left({t}_{0}\right)}{dt}&=\frac{1}{S({t}_{0})}{\int_{{t}_{0}}^{{{\infty }}}}{td}\left[-S\left(t\right)\right]\\&={\left.-\frac{1}{S({t}_{0})}{tS}\left(t\right)\right|}_{{t}_{0}}^{{{\infty }}}+\frac{1}{S({t}_{0})}{\int_{{t}_{0}}^{{{\infty }}}}S(t){dt}\\& ={t}_{0}+\frac{1}{S({t}_{0})}{\int_{{t}_{0}}^{{{\infty }}}}S\left(t\right){dt}.\end{array}$$

However, the conditional expectation given in the theorem may not be an appropriate label for the machine learning algorithm. The formula does not consider the confidence of the estimation; it will always return a value greater than the mean survival and have a bias toward the long survival class. To address this problem, we estimate the survival as follows:

$${\mathrm{{survival}}}=\left\{\begin{array}{c}{\mathrm{{mean}}},\,{\mathrm{if}}\,{t}_{0}\le \frac{{\mathrm{{mean}}}}{2}\\ {t}_{0}+\frac{1}{S\left({t}_{0}\right)}{\int_{{t}_{0}}^{{{\infty }}}}S\left(t\right){dt},\,{\mathrm{if}}\,{t}_{0}\, > \,\frac{{\mathrm{{mean}}}}{2}\end{array}\right.$$

To select biomarkers for the prediction of survival groups, we used a naïve Bayesian classifier. However, Bayesian classifiers suffer from severe numerical underflow problems when the dimension of the data is high. Even with careful scaling, all but the dominant feature is still likely to underflow. To solve this problem, we developed a generalized naïve Bayesian classifier by applying a geometric mean to the likelihood product. We prove that this approach eliminates the underflow problem, and the geometric mean is essentially the only function satisfying these conditions.

The naïve Bayesian classifier is a simple but often effective machine learning algorithm. It is based on Bayes’ theorem and the assumption that all attributes are conditionally independent.

Let $({x}_{1},{x}_{2},\ldots ,{x}_{d})$ be the input attribute vector and $({C}_{1},{C}_{2},\ldots ,{C}_{k})$ be the classes. According to Bayes Theorem,

$$P\left({C}_{j}|{x}_{1},{x}_{2},\ldots ,{x}_{d}\right)=\frac{P({C}_{j})P\left({x}_{1},{x}_{2},\ldots ,{x}_{d},|,{C}_{j}\right)}{\mathop{\sum }\nolimits_{i=1}^{k}P({C}_{i})P\left({x}_{1},{x}_{2},\ldots ,{x}_{d},|,{C}_{i}\right)}.$$

With the assumption of conditional independence, we have

$$P\left({x}_{1},{x}_{2},\ldots ,{x}_{d},|,{C}_{j}\right)=\,P\left({x}_{1}{\rm{|}}{C}_{j}\right)P\left({x}_{2}{\rm{|}}{C}_{j}\right)\ldots P\left({x}_{d}{\rm{|}}{C}_{j}\right).$$

The probabilities $P\left({x}_{i}|{C}_{j}\right)$ can be easily estimated from training data. However, when dimension *d* is large, the products of the probabilities (likelihood) become extremely small, causing underflows. If each probability value has an average of 1/2, the likelihood will have a mean

$$E\left[P\left({x}_{1}{\rm{|}}{C}_{j}\right)P\left({x}_{2}{\rm{|}}{C}_{j}\right)\ldots P\left({x}_{d}{\rm{|}}{C}_{j}\right)\right]=\,\frac{1}{{2}^{d}},$$

which approaches 0 quickly when *d* is large.

One typical method to avoid numerical underflow is to scale all the values using the largest probability product during the computations. However, this method often produces one value that dominates the probability products. As a result, one class will have a predicted probability of 1.0 while all other classes will have a prediction probability of 0.0. This effect is disadvantageous for most applications because it is an artifact of the naïve Bayesian assumption and usually does not reflect the real probability.

We propose a generalization to the standard naïve Bayesian algorithm to address the underflow problem. Let h(x) be a positive increasing function. Applying the function to the likelihood produces a new probability estimate:

$$P\left({x}_{1},{x}_{2},\ldots ,{x}_{d},|,{C}_{j}\right)=h[P\left({x}_{1}{\rm{|}}{C}_{j}\right)P\left({x}_{2}{\rm{|}}{C}_{j}\right)\ldots P\left({x}_{d}{\rm{|}}{C}_{j}\right)].$$

In particular, we propose to use the function

$$h\left(x,d\right)={x}^{1/d},$$

which increases monotonically with *d* and prevents underflow for any dimension d.

#### **Lemma**

Let *x* be a uniform random value over the interval [0, 1]; the expected value of *x* $h\left(x,d\right)={x}^{1/d}$ for a constant *d* is $\frac{1}{{(1+1/d)}^{}}$.

#### Proof

Because x is uniform, the expected value of ${x}^{1/d}$ is

$${\int_{0}^{1}}{x}^{1/d}{dx}={\left.\frac{{x}^{1+1/d}}{1+1/d}\right|}_{0}^{1}=\frac{1}{{(1+1/d)}}.$$

#### Theorem

Assume that the probabilities in the likelihood are independent, uniformly distributed random variables. Then, the expected value of the likelihood is

$$E\left[{(P\left({x}_{1}{\rm{|}}{C}_{j}\right)P\left({x}_{2}{\rm{|}}{C}_{j}\right)\ldots P\left({x}_{d}{\rm{|}}{C}_{j}\right))}^{1/d}\right]=\,\frac{1}{{(1+1/d)}^{d}}.$$

#### Proof

By the previous lemma and the independence of the random variables,

$$\begin{array}{ll}E\left[{\left(P\left({x}_{1}{\rm{|}}{C}_{j}\right)P\left({x}_{2}{\rm{|}}{C}_{j}\right)\ldots P\left({x}_{d}{\rm{|}}{C}_{j}\right)\right)}^{\frac{1}{d}}\right]\\ \quad=E\left[{\left(P\left({x}_{1}{\rm{|}}{C}_{j}\right)\right)}^{\frac{1}{d}}\right]E\left[{\left(P\left({x}_{2}{\rm{|}}{C}_{j}\right)\right)}^{\frac{1}{d}}\right]\ldots E\left[{\left(P\left({x}_{d}{\rm{|}}{C}_{j}\right)\right)}^{\frac{1}{d}}\right]=\,\frac{1}{{(1+1/d)}^{d}}.\end{array}$$

The limit of the expected value is

$$\mathop{{\rm{lim}}}\limits_{d\to {\rm{\infty }}}\frac{1}{{(1+1/d)}^{d}}=1/e.$$

Therefore, as the dimension increases, the likelihood will never approach 0 uniformly. Applying the function *h* to the likelihood does not change the relative order of the probability estimates of the classes. However, the probabilities will have more reasonable values than 0 and 1.

We can also show that the function $h\left(x,d\right)={x}^{1/d}$ is unique under certain conditions.

#### Lemma

Let $f\left(x\right)$ be a positive continuous function of positive real numbers. If *f* is multiplicative, $f\left({xy}\right)=f\left(x\right)f(y)$, then $f\left(x\right)={x}^{a}$ for some constant *a*.

In the case of the functional transform on the likelihood, the assumption of the multiplicative property on the function *h* is a natural extension of the naïve Bayesian assumption.

If we require that the likelihood approaches a non-zero limit as *d* approaches infinity, then the function could have the form $h\left(x,d\right)={x}^{c/d}$ for a constant *c*.

#### Theorem

If *h* is multiplicative and

$$\mathop{{\rm{lim}}}\limits_{d\to {{\infty }}}E\left[h\left(P\left({x}_{1}{\rm{|}}{C}_{j}\right)P\left({x}_{2}{\rm{|}}{C}_{j}\right)\ldots P\left({x}_{d}{\rm{|}}{C}_{j}\right)\right)\right]=L\, > \,0,$$

then $$h\left(x,d\right)={x}^{a(d)}$$, where $$a\left(d\right)=c\left(\frac{1}{d}\right)+O\left(\frac{1}{{d}^{2}}\right),c \;>\; 0$$.

#### Proof

The previous lemma shows that

$$h\left(x,d\right)={x}^{a(d)}.$$

Similar to the previous proof, the expectation is

$$\begin{array}{ll}E\left[h\left(P\left({x}_{1}{\rm{|}}{C}_{j}\right)P\left({x}_{2}{\rm{|}}{C}_{j}\right)\ldots P\left({x}_{d}{\rm{|}}{C}_{j}\right)\right)\right]\\ \quad\;=E\left[{\left(P\left({x}_{1}{\rm{|}}{C}_{j}\right)P\left({x}_{2}{\rm{|}}{C}_{j}\right)\ldots P\left({x}_{d}{\rm{|}}{C}_{j}\right)\right)}^{a(d)}\right]\\ \quad\;=E\left[{\left(P\left({x}_{1}{\rm{|}}{C}_{j}\right)\right)}^{a(d)}\right]E\left[{\left(P\left({x}_{2}{\rm{|}}{C}_{j}\right)\right)}^{a(d)}\right]\ldots E\left[{\left(P\left({x}_{d}{\rm{|}}{C}_{j}\right)\right)}^{a(d)}\right]\\ \quad\;=\frac{1}{{(1+a(d))}^{d}}.\end{array}$$

By the assumption, we have

$$\mathop{{\rm{lim}}}\limits_{d\to {{\infty }}}\frac{1}{{(1+a\left(d\right))}^{d}}=L \,>\, 0.$$

Letting$\,t=1/d$ and $f\left(t\right)=a\left(1/t\right)=a(d)$, then

$$\mathop{{\rm{lim}}}\limits_{d\to {{\infty }}}\frac{1}{{\left(1+a\left(d\right)\right)}^{d}}=\mathop{{\rm{lim}}}\limits_{t\to 0+}\frac{1}{{\left(1+f\left(t\right)\right)}^{\frac{1}{t}}}=\mathop{{\rm{lim}}}\limits_{t\to 0+}{e}^{\frac{-{\rm{ln}}(1+f\left(t\right))}{t}}.$$

Furthermore, $$f\left(0\,+\right)=0$$ and

$$\mathop{{\rm{lim}}}\limits_{t\to 0}{e}^{\frac{-{\rm{ln}}(1+f\left(t\right))}{t}}=\mathop{{\rm{lim}}}\limits_{t\to 0}{e}^{\frac{-{f}^{{\prime} }(t)}{1\,+\,f\left(t\right)}}=\mathop{{\rm{lim}}}\limits_{t\to 0}{e}^{-{f}^{{\prime} }(t)}={e}^{-c}=L.$$

Therefore,

$${f\left(t\right)={ct}+O(t}^{2}),$$

$$a\left(d\right)=c\left(\frac{1}{d}\right)+O\left(\frac{1}{{d}^{2}}\right),c\, > \,0.$$

When the dimension *d* is high, the independence assumption of the naïve Bayesian classifier is unlikely to be true in most applications. Consequently, the probability estimates are unrealistic. Our proposed extension can solve this problem.

#### Example

Consider a two-class problem with d-dimensional Gaussian distributions, with means of

$\left(\mathrm{1,1},\ldots ,1\right)$ and $\left(-1,-1,\ldots ,-1\right)$ and the same covariance matrix

$$\left[\begin{array}{cccc}1 & r & \cdots & r\\ r & 1 & \cdots & r\\ \vdots & \vdots & \ddots & \vdots \\ r & r & \cdots & 1\end{array}\right]=\left(1-r\right)I+{rJ};$$

the inverse matrix is

$$\frac{1}{1-r}\left(I-\frac{r}{1-r+{rd}}J\right).$$

Consider the probability estimations for the point $\left(t,t,\ldots ,t\right)$. The true probability for class 1 is

$$\frac{{e}^{-0.5d{(t\,-\,1)}^{2}\left(1-\frac{{rd}}{1\,-\,r\,+\,{rd}}\right)}}{{e}^{-0.5d{(t\,-\,1)}^{2}\left(1\,-\,\frac{{rd}}{1\,-\,r\,+\,{rd}}\right)}+{e}^{-0.5d{(t+1)}^{2}\left(1-\frac{{rd}}{1\,-\,r\,+\,{rd}}\right)}}$$

For the original naïve Bayesian classifier,

$$\frac{{e}^{-0.5d{(t\,-\,1)}^{2}}}{{e}^{-0.5d{(t\,-\,1)}^{2}}+{e}^{-0.5d{(t\,+\,1)}^{2}}},$$

and for our proposed classifier,

$$\frac{{e}^{-0.5{(t\,-\,1)}^{2}}}{{e}^{-0.5{(t\,-\,1)}^{2}}+{e}^{-0.5{(t\,+\,1)}^{2}}}.$$

Figure S3 shows the three probability estimates for *d* = 10 and *r* = 0.5. The naïve Bayesian probability estimates change steeply around the boundary owing to the independence assumption. In contrast, our proposed method closely approximates the true probabilities.

### Feature selection

We used a discriminant measure for single genes to facilitate gene selection. This method was based on cross-validation to avoid overfitting. This measure is consistent with the generalized naïve Bayesian classifier. To fully utilize the survival data, we used a parameter estimation method on the means and variations for the generalized naïve Bayesian classifier. By modeling the relationship between survival time and classes, we obtained an improved formula for estimating the means and variances of the distributions.

A single level of gene selection and classification for this survival analysis problem is not adequate for detecting groups defined by NGS biomarkers. Thus, a hierarchical approach was developed to use multiple levels of gene selection and classification for the prediction of survival as well as the detection of biomarker-related groups. Owing to the inherent uncertainties in the survival data, it is usually not feasible to include a large number of genes in machine learning algorithms. Thus, a subset of genes relevant to the prediction task was selected.

Standard dimension reduction methods, such as principal component analysis (PCA) and recursive feature elimination, start with a system with all features included. It would be difficult to obtain effective features from noisy survival data in such a highly over-fitted and volatile system. In PCA-based methods, it is also difficult to extract an explicit gene list because the mappings would involve the entire set of genes. Following the same principle applied in the naïve Bayesian classifier, we propose a feature selection method to select and rank genes based on a discriminant measure of individual genes.

To reduce the effects of noise and avoid overfitting, we employ k-fold cross-validation to obtain a robust measure. For an individual gene, a generalized naïve Bayesian classifier was constructed on the training subset and tested on the testing subset. The complement ${d}_{12}$ of the cross-validation error rate was used as a discriminant measure for the gene.

$$\,{d}_{12}=1-{{{\mathrm{error}}}}_{12}$$

The genes were ranked by ${d}_{12}$; higher values corresponded to more relevant genes for classifying the two classes. The survival data consisted of continuous values that did not represent a class label directly; however, the magnitude of the values provide useful information on the class. We estimated the mean and variance of the distribution in the generalized naïve Bayesian classifier by weighted averages based on the relationship between survival time and class membership.

Let $y$ be the survival time and $P({C}_{k}{|y})$ be the conditional probability function connecting ${y}$ and class ${C}_{k}$. Assuming that there are two classes and $P\left({{y|C}}_{k}\right),{k}=\mathrm{1,2}$ are Gaussian with equal variances, according to Bayes’ theorem,

$$P\left({C}_{k}|y\right)=\frac{P\left({y{\rm{|}}C}_{k}\right)P\left({C}_{k}\right)}{P\left({y{\rm{|}}C}_{1}\right)P\left({C}_{1}\right)+P\left({y{\rm{|}}C}_{2}\right)P\left({C}_{2}\right)}=\frac{1}{1+{e}^{a(y-b)}},$$

which is a logistic function.

Given the training cases $\left({x}_{i},{y}_{i}\right),{i}={1,2},\ldots ,n$, we have the likelihood function

$$L=-\mathop{\sum }\limits_{i=1}^{n}{\rm{ln}}\left[\mathop{\sum }\limits_{k=1}^{2}P({C}_{k}{\rm{|}}y)P({x}_{i}{\rm{|}}{C}_{k})\right].$$

Maximizing the likelihood, we obtained

$$\frac{\partial L}{\partial {m}_{k}}=\mathop{\sum }\limits_{i=1}^{n}\frac{P({C}_{k}|{y}_{i})P({x}_{i}|{C}_{k})}{\mathop{\sum }\nolimits_{k=1}^{2}P({C}_{k}|{y}_{i})P({x}_{i}|{C}_{k})}({x}_{i}-{m}_{k})=0.$$

The coefficients involve unknown values $P\left({x}_{i},|,{C}_{k}\right)$. If they are set as constants, we can solve the equations and obtain an explicit formula for the means:

$${m}_{k}=\mathop{\sum }\limits_{i=1}^{n}\frac{P\left({C}_{k}|{y}_{i}\right){x}_{i}}{{\mathop{\sum}\nolimits_{j=1}^{n}}P({C}_{k}|{y}_{j})}={\mathop{\sum}\limits_{i=1}^{n}}{w}_{i}{x}_{i},$$

The weighted average is ${x}_{i}$. The weights are proportional to the class probability on ${y}_{i}$:

$${w}_{i}=\frac{P\left({C}_{k}|{y}_{i}\right)}{\mathop{\sum}\nolimits_{j=1}^{n}P({C}_{k}|{y}_{j})}.$$

Similarly, the variances can be estimated as follows:

$${{\sigma }^{\,}}_{k}^{2}=\mathop{\sum }\limits_{i=1}^{n}\frac{P\left({C}_{k}|{y}_{i}\right){(x_{i}-{m}_{k})}^{2}}{\mathop{\sum }\nolimits_{j=1}^{n}P({C}_{k}|{y}_{j})}=\mathop{\sum }\limits_{i=1}^{n}{w}_{i}{(x_{i}-{m}_{k})}^{2}.$$

## Supplementary information

Supplemental file
