# Supplementary material for: Determining clinical course of diffuse large B-cell lymphoma using targeted transcriptome and machine learning algorithms
Source: Blood Cancer J. 2022 Feb 1;12(2):25. doi: 10.1038/s41408-022-00617-5 (PMC8807629; doi:10.1038/s41408-022-00617-5)
Supplement: Supplementary file 1 — Supplemental file [file 41408_2022_617_MOESM1_ESM.docx]

**Supplementary Material**

**Supplementary Figures**

**
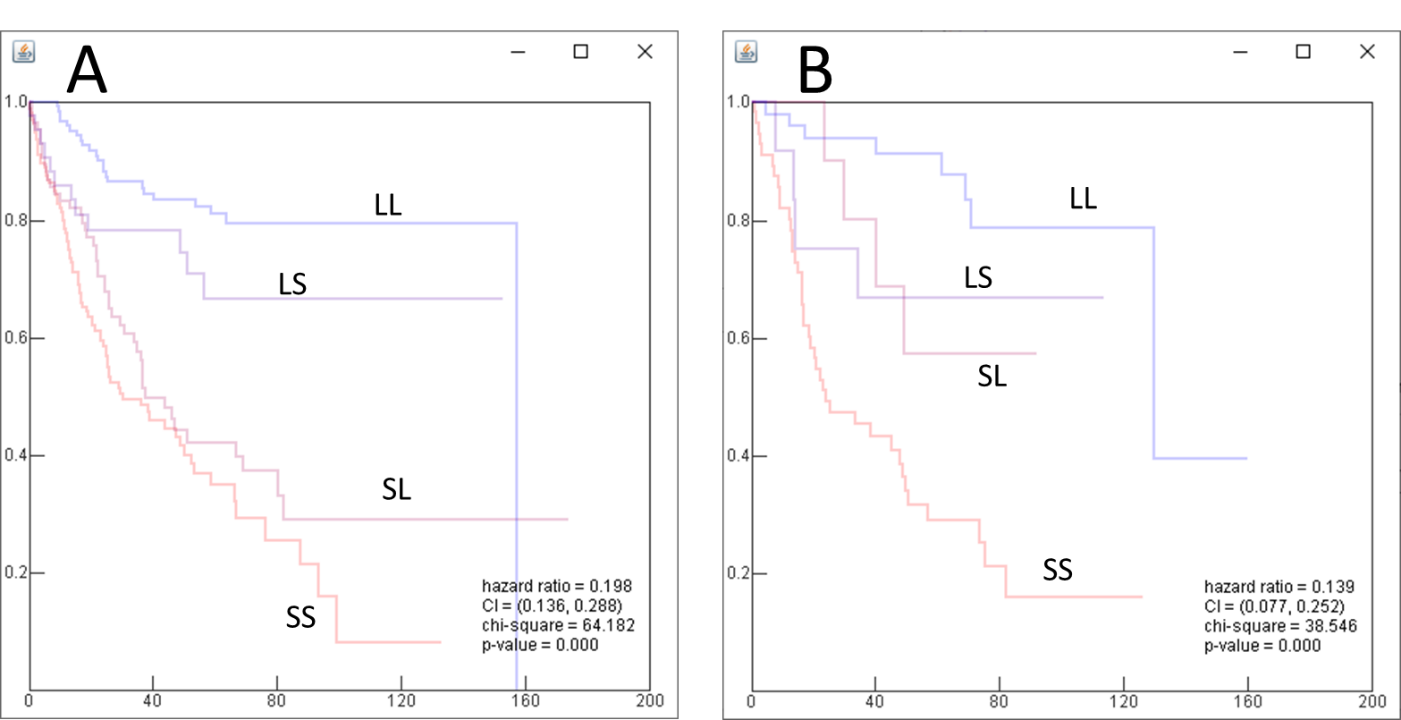
**

**Fig. S1. Alternative survival model** combining patients (626 patients), then using two-thirds for building the model (training) (Panel A) and one-third for testing (panel B).

**
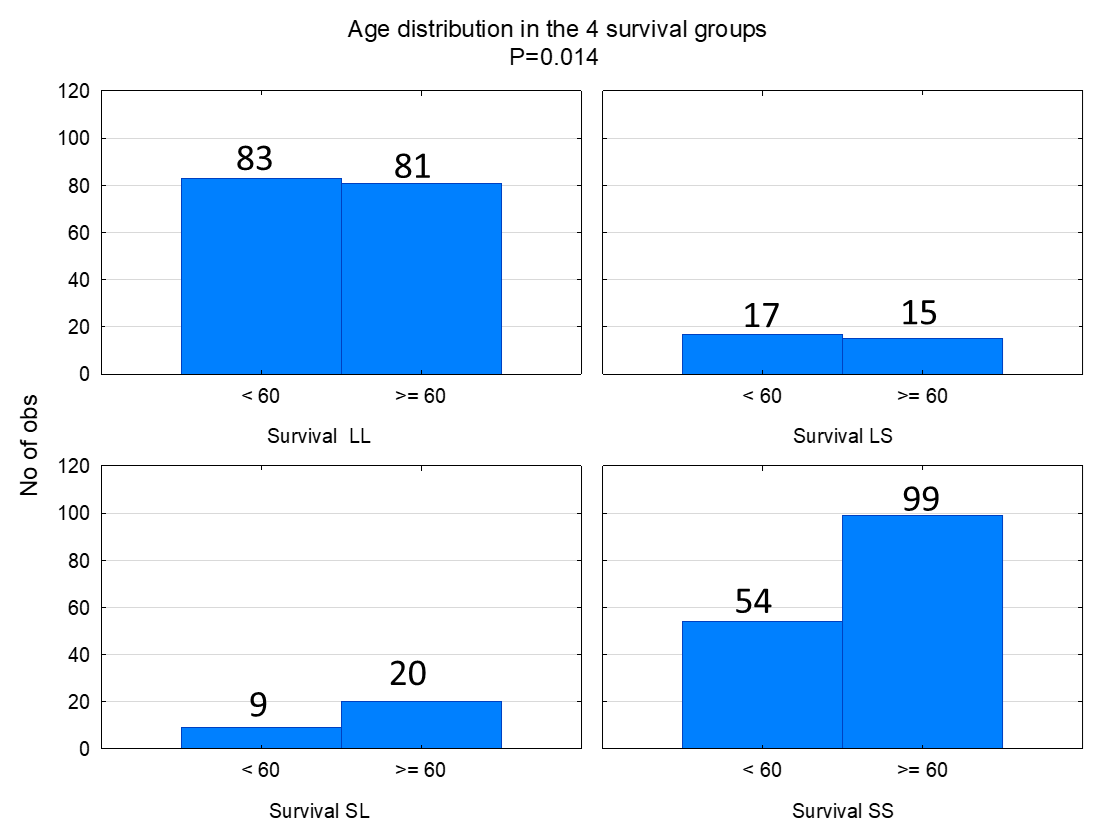
**

**Fig S2.** Poor survival subgroup (SS) shows significantly (P = 0.01) higher percentage of patients at age above 60.

**
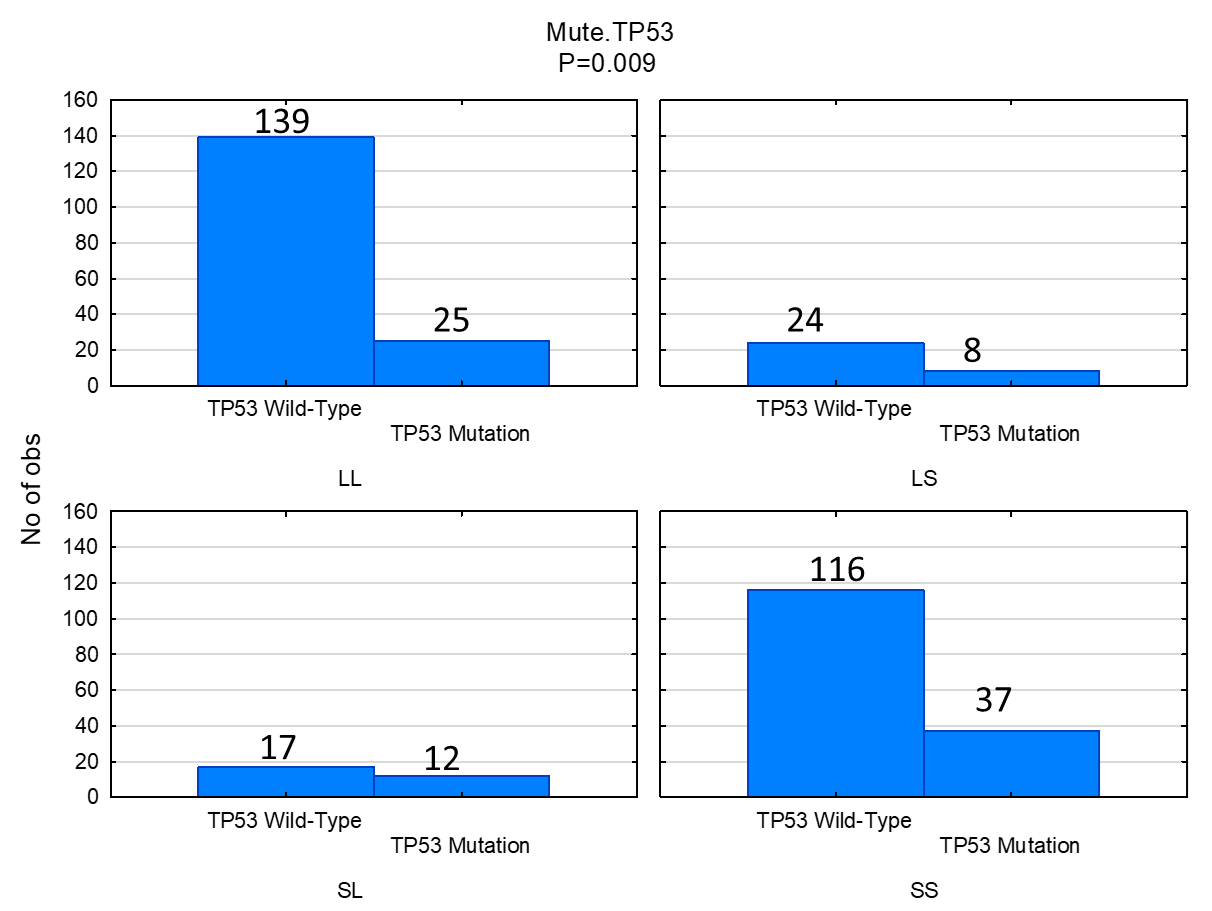
**

**Fig. S3. TP53 mutation as predictor of survival**

**
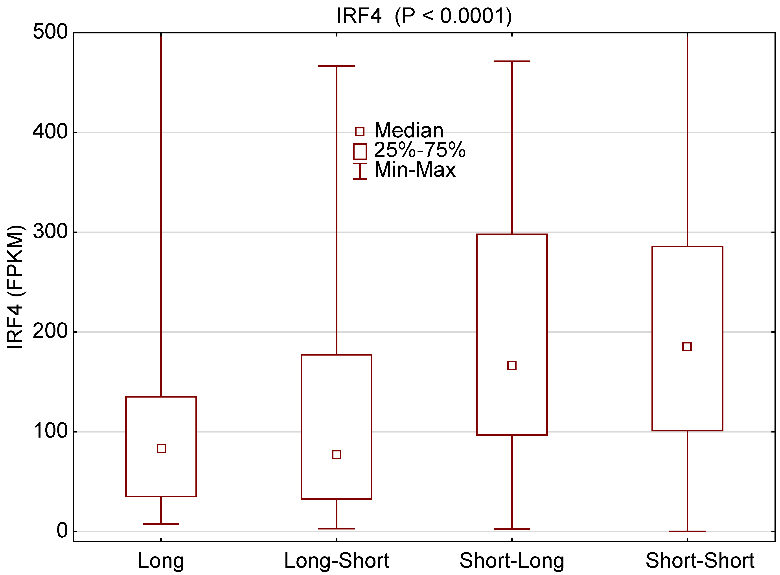
**

**Fig. S4. IRF4 overexpression as predictor of survival**

**
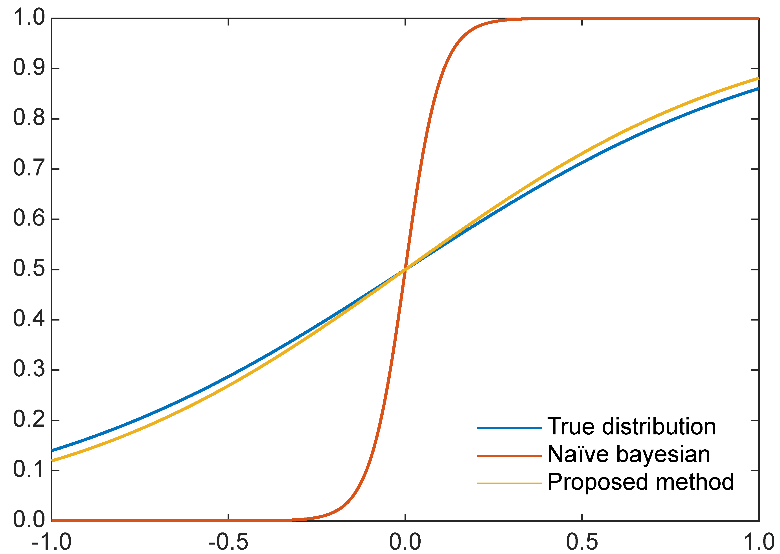
**

**Fig. S5. Smoothing Bayesian prediction score to facilitate a comparison between each biomarker.**

**Supplementary Table**

**Table S1**. RNA biomarkers selected using the modified naïve Bayesian classifier and cross validation for the prediction of survival

| **Long survival vs short survival** | | | | | | | | | |
| --- | --- | --- | --- | --- | --- | --- | --- | --- | --- |
| PPP2R1B | GOLGA5 | LINGO2 | HMGA1 | SIN3A | ARID1A | BCL7A | CDK5RAP2 | MAGED1 | CREB3L1 |
| AMER1 | DLL1 | GSTT1 | GPR34 | DNM2 | CCNB1IP1 | MUTYH | RET | CDH1 | POFUT1 |
| XRCC6 | KIT | RALGDS | SS18 | CD22 | BRCA2 | HDAC3 | LHX4 | FAM19A2 | PRG2 |
| PRCC | TBL1XR1 | HIF1A | EDIL3 | ROS1 | DKK4 | CDC25A | WNT7B | MYBL1 | MLLT10 |
| SLCO1B3 | TACC2 | CANT1 | NCAM1 | FGF3 | FGF19 | PPP3R2 | CRADD | ETV6 | SPP1 |
| SDHB | FGF2 | SUZ12 | MB21D2 | MYC | BAX | CEP57 | ITGA5 | ABCC3 | HECW1 |
| **Long vs short survival in long survival group** | | | | |  |  |  |  |  |
| DUSP22 | CTNNA1 | DUX2 | SSX1 | SSX2 | CTNNB1 | DCLK2 | FH | DUSP9 | FCGR2B |
| STAT5B | ESR1 | CD274 | TERF1 | AKAP9 | DGKI | HMGA1 | ARNT | MAFB | PPP3CC |
| COL3A1 | NUTM2A | CIT | MGMT | CDK6 | SORT1 | RCSD1 | CDK5RAP2 | SIN3A | RABEP1 |
| MB21D2 | KDR | SS18L1 | SSBP2 | SH2D5 | ASXL1 | AMER1 | AFF1 | PRKCD | 2-Sep |
| TPM4 | FIGF | NODAL | GRM3 | STAT6 | GAB1 | RPL22 | BDNF | SNX29 | MELK |
| ARRDC4 | FGF10 | MMP9 | YY1AP1 | HAS2 | DLEC1 | DEK | TLL2 | BCL2L2 | ID3 |
| **Long vs short survival in short survival group** | | | | |  |  |  |  |  |
| AHI1 | EPHA5 | DUSP22 | DUSP26 | DUSP9 | DUX2 | MGMT | MIB1 | MIPOL1 | MIR1260B |
| MIR4321 | MIR4683 | MIR4758 | MIR6515 | MIR6752 | MIR6765 | BIVM-ERCC5 | SSX1 | SSX2 | LTBP1 |
| MAFB | TLR4 | CTNNB1 | ETV5 | CHEK2 | FUS | SS18L1 | SSBP2 | DGKI | CIT |
| TFE3 | FGF19 | TRIM33 | CTCF | LAMA1 | TBL1XR1 | TOP1 | RB1 | OLR1 | DOCK1 |
| ARID1A | RABEP1 | EP400 | STK11 | ETS1 | MAPK1 | CDC14A | LMO7 | SS18 | ICK |
| FLI1 | POU5F1 | RCSD1 | HRAS | BACH2 | CDK7 | GAS5 | CARS | SRSF2 | MAP3K6 |

| Table S2. Patient Characterstics | |
| --- | --- |
| Characterstics | Number (%) |
| No. of patients | 379 |
| Age |  |
| <60 | 164 (43%) |
| ≥60 | 215 (57%) |
| Sex |  |
| Male | 210 (55%) |
| Female | 169 (45% |
| Cell of origin |  |
| GCB | 198 (52%) |
| ABC | 181 (48%) |
| Stage |  |
| I-II | 185 (49%) |
| III-IV | 194 (51) |
| IPI |  |
| ≤2 | 238 (63%) |
| >2 | 141 (37% |
| ECOG |  |
| ≤ 1 | 319 (84%) |
| >1 | 60 (16%) |
| LDH |  |
| Normal | 162 (43%) |
| High | 217 (57% |
